# Supplementary material for: Chemoresistant Cancer Cell Lines Are Characterized by Migratory, Amino Acid Metabolism, Protein Catabolism and IFN1 Signalling Perturbations
Source: Cancers (Basel). 2022 Jun 2;14(11):2763. doi: 10.3390/cancers14112763 (PMC9179525; doi:10.3390/cancers14112763)
Supplement: Supplementary file 1 [file cancers-14-02763-s001.zip › cancers-1716338-SI.pdf]

A.

Patient	Age at diagnosis	Stage at diagnosis	Diagnosis	Chemo resistant Status
P1	61	3c	Serous carcinoma of the ovary	Sensitive
P2	43	?	Recurrent chemotherapy resistant ovarian carcinoma	Resistant

B.

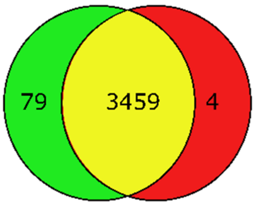

C.

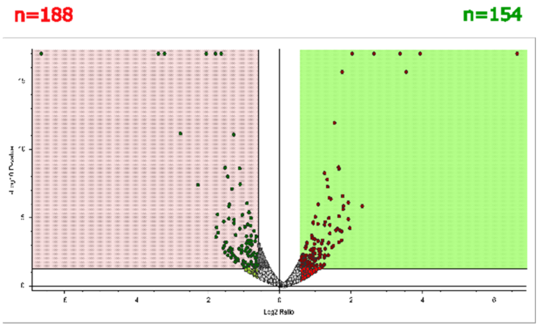

D.

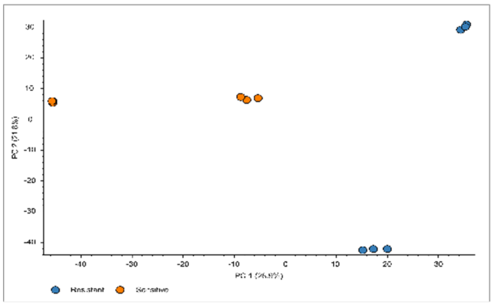

E.

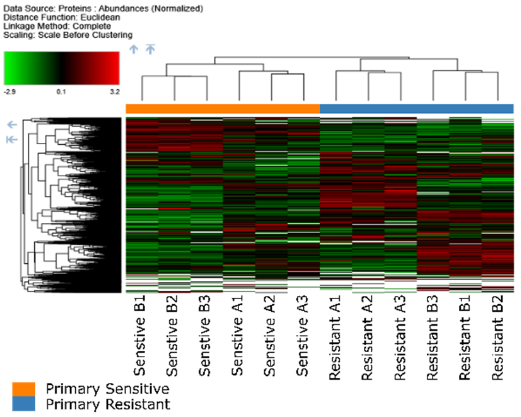

F.

Rank	Term	Count	Involved genes/total genes (%)	P-value
1	type I interferon signaling pathway	10	3.1	7.40E-06
2	mitochondrial translation	12	12	1.10E-05
3	oxidation-reduction process	38	38	2.10E-05
4	response to oxidative stress	21	21	3.60E-05
5	cellular component disassembly	23	23	1.30E-04
6	Generation of precursor metabolites and energy	19	5.8	7.60E-02
7	carboxylic acid metabolic process	31	9.5	7.60E-02
8	protein complex subunit organization	51	15.7	7.60E-02
9	single-organism catabolic process	31	9.5	7.60E-02
10	NAD metabolic process	7	2.2	1.60E-01

Supplementary Figure S1: Proteomics investigation of primary ovarian cancer samples taken from chemosensitive (n=1) and chemoresistant (n=1). (**A**) Table of patient details for P1 (chemosensitive) and P2 (chemoresistant) primary cell samples. (**B**) Venn diagram of proteins identified in primary samples showing 3459 proteins identified in common between primary samples (P1 total=3538, P1 exclusive=79, P2 total=3463, P2 exclusive=4, total proteins identified=3542). (**C**) Volcano plot of abundance ratios (resistant/sensitive) using a 1.5-fold cut off (0.58 log2, $p<0.05$) shows 188 proteins more abundant in P2 (resistant) and 154 proteins more abundant in P1 (sensitive). (**D**) Principle component analysis shows clear separation of primary cells based on their origin. E) Hierarchical clustering shows clear separation of primary cells based on their cell origin. (**F**) Table of the top 10 gene ontology biological functions related to 1.5 fold differentially abundant proteins between P1 and P2. Analysis performed via the DAVID functional gene annotation platform and duplicate categories and subcategories removed.

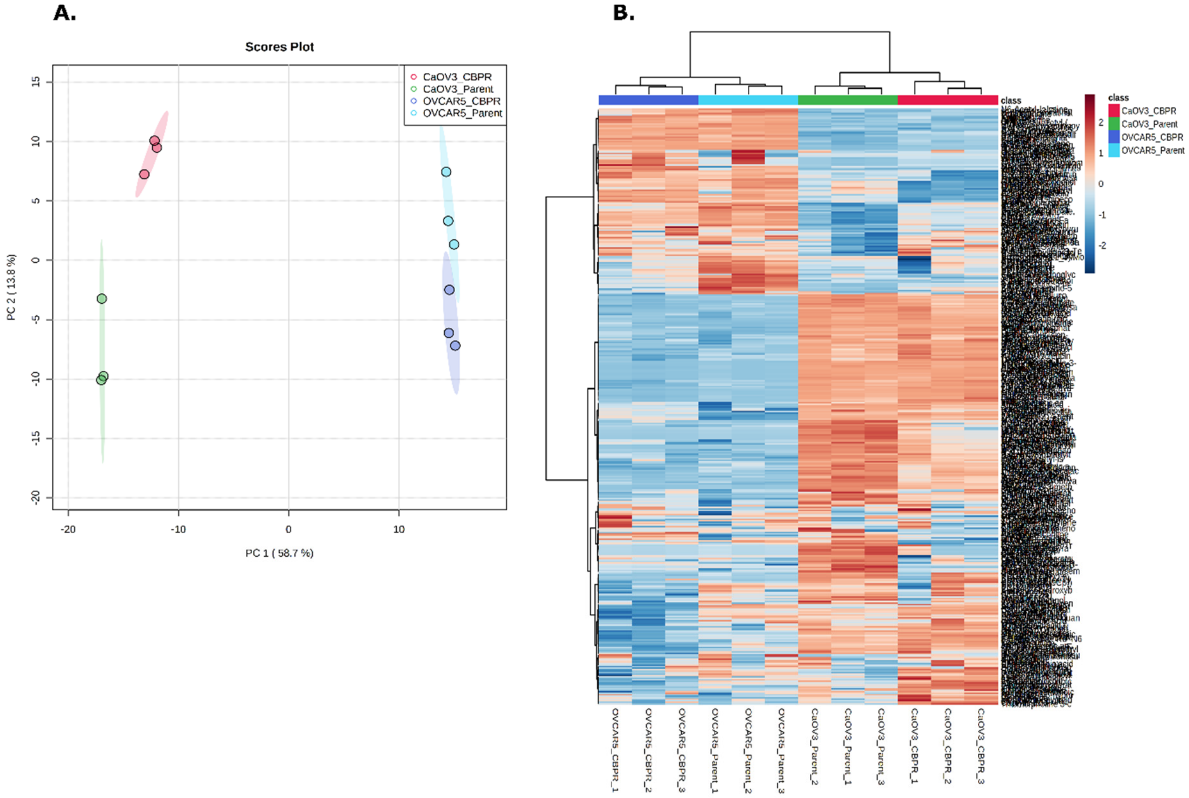

Supplementary Figure S2: PCA and hierarchical clustering of all metabolites across both OVCAR-5 and CaOV3 Parental and CBPR cell lines. (**A**) PCA scores plot shows separation based on chemoresistant status within cell lines but significantly larger separation between cell lines. (**B**) Hierarchical clustering of all metabolites across all cell lines shows clear separation based on chemoresistant status within cell lines. Shows a larger separation between different cell lines regardless of chemoresistance status.

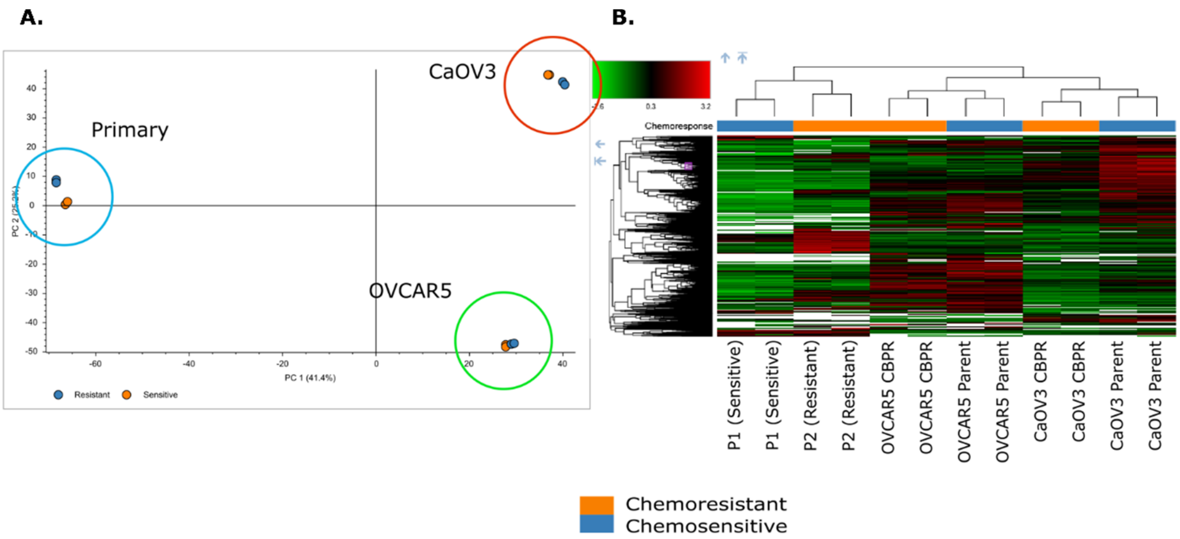

Supplementary Figure S3: PCA and hierarchical clustering of all proteins across Parental and CBPR cell lines and chemosensitive and chemoresistant primary cells. (**A**) PCA plot shows clear separation of proteomic features based on sample origin. (**B**) Hierarchical clustering of all proteomes shows clear clustering of each sample type. Further clustering observed based on cell origin.
